# A 5 years’ experience of a parent-baby day unit: impact on baby’s development

**DOI:** 10.3389/fpsyt.2023.1121894

**Published:** 2023-06-15

**Authors:** Audrey Moureau, Louise Cordemans, Caroline Gregoire, Pirmez Benoît, Veronique Delvenne

**Affiliations:** ^1^Faculty of Medicine, Université Libre de Bruxelles, Brussels, Belgium; ^2^Child and Adolescent Psychiatry Department, Queen Fabiola Children’s University Hospital, Brussels, Belgium; ^3^Faculty of Psychology, Université Libre de Bruxelles, Brussels, Belgium; ^4^Faculty of Statistics, Université Catholique de Louvain, Louvain-la-Neuve, Belgium

**Keywords:** perinatality, mother-baby unit, parent-baby day unit, day treatment, child development, parent-child relation

## Abstract

**Introduction:**

Psychiatric Mother-Baby Units are well established in France, United Kingdom, and Australia, mostly in full-time hospitalization. Inpatient units are considered as best practice for improving outcomes for mothers and babies when the mother is experiencing severe mental illness and many studies have showed the effectiveness of care for the mother or the mother-infant relationship. Only a limited number of studies have focused on the day care setting or on the development of the baby. Our parent-baby day unit is the first day care unit in child psychiatry in Belgium. It offers specialized evaluation and therapeutic interventions focused on the baby and involves parents with mild or moderate psychiatric symptoms. The advantages of day care unit is to reduce the rupture with social and family living.

**Aims:**

The objective of this study is to evaluate the effectiveness of parent-baby day unit in prevention of babies’ developmental problems. First, we present the clinical characteristics of the population treated in the day-unit in comparison to the features presented in the literature review about mother-baby units, which usually receive full-time treatment. Then, we will identify the factors that might contribute to a positive evolution of the baby’s development.

**Materials and methods:**

In this study, we retrospectively analyze data of patients admitted between 2015 and 2020 in the day unit. Upon admission, the 3 pillars of perinatal care – babies, parents, and dyadic relationships – have systematically been investigated. All the families have received a standard perinatal medico-psycho-social anamnesis, including data on the pregnancy period. In this unit, all the babies are assessed at entry and at discharge using the diagnostic 0 to 5 scale, a clinical withdrawal risk, and a developmental assessment (Bayley). Parental psychopathology is assessed with the DSM5 diagnostic scale and the Edinburgh scale for depression. Parent–child interactions are categorized according to Axis II of the 0 to 5 scale. We have evaluated the improvement of children symptomatology, the child development and the mother–child relation between the entrance (T1) and the discharge (T2) and we have compared two groups of clinical situations: a group of patients with a successful evolution (considering baby’s development and the alliance with the parents) and a group of unsuccessful evolution during hospitalization.

**Statistical analysis:**

We use descriptive statistics to characterize our population. To compare the different groups of our cohort, we use the *T*-test and non-parametric tests for continue variables. For discrete variables, we used the Chi^2^ test of Pearson.

**Discussion:**

The clinical population of the day unit is comparable to the mother-baby units in terms of psychosocial fragility but the psychopathological profile of the parents entering the day unit shows more anxiety disorder and less post-partum psychosis. The babies’ development quotient is in the average range at T1 and is maintained at T2. In the day unit, the number of symptoms as well as the relational withdrawal of the babies is reduced between T1 and T2. The quality of parent–child relationship is improved between T1 and T2. The children of the group of pejorative evolution had a lower developmental quotient at the T1 and an overrepresentation of traumatic life events.

**Conclusion:**

These results indicate that parent-baby day unit lead to positive outcomes in clinical situations with anxio-depressive parents, relational withdrawal of the babies, functional problems of the babies but not when a significant impact on the development of the baby already exists. The results of this study can guide therapeutic approaches for the benefit of care in parent-baby day units, and improve the development of the child and of the dyadic relationships.

## Introduction

In western countries, perinatal mental disorders are associated with considerable maternal and fetal/child morbidity and mortality which remain one of the major problem for the child development and adaptability (1–3). Childhood toxic stress is defined as a severe, prolonged, or repetitive adversity, and a lack of the nurturance or support from a caregiver. It induces a disruption of the neuro-endocrine-immune response resulting in prolonged cortisol activation and a persistent inflammatory state. Children experiencing early life toxic stress are at risk of long-term adverse health effects including maladaptive coping skills, emotional, social and cognitive long-lasting effects, poor stress management, unhealthy lifestyles, mental illness, and physical disease (4–8). Some of the negative effects of early life toxic stress on child development are mediated by disruptions in the mother–child interaction, biological factors including cortisol secretion and epigenetic processes (3, 9–12).

Furthermore, in the context of early distorted parent–child interactions, number of studies have shown that treatment targeting only the mother is not sufficient to improve the development of the child (9, 13).

Additionally, the risk factors for parental psychiatric troubles are also risk factors for poor child development, suggesting that the relation between parental disorder and compromised early child development is multilevel and cumulative (14).

Regarding maternal depression, which is to date the most widely studied perinatal pathology, many authors conclude the need for care integrating as soon as possible the parent, the child and their emerging relationship to reduce the impact of cortisol secretion on the development of the child (15, 16).

Parent-baby units are an interesting perinatal care device recommended by the NICE (National institute for Health and Care Excellence) for women with acute postpartum mental disorders and their baby to facilitate mother-infant relationships (1). These units provide health care for mothers, care for the infant and sustain the mother-infant interactions (17). This type of unit has been established in several countries since the eighties (United Kingdom, France, and Australia) and more recently in New Zealand, Israel, India, Sri-Lanka, and in the US (18). In 2021 in France, following the publication of the “First 100 Days” report, additional budget was allocated for the opening of 10 new mother-baby units. This report also points out the difficulty to compare the different units, due to the heterogeneity of the existing devices (19, 20). First, the literature still refers to “mother-baby units,” despite the fact that fathers are more frequently involved. Second, some parent-baby or mother-baby unit are day-night units, other are day units. Third, these units depend, based on their funding or their professional identity, either of adult psychiatry or of child psychiatry department.

Day units were created in parallel to day-night units (21). The choice of implementing a day structure has being often induced by economic necessity (22). These structures are positioned at an intermediate level for dyadic care, between outpatient care and full-time hospitalization. So, in case of parental psychopathology, it has to be stabilized and it should not compromise too much the parenting function at home.

Most studies on mother-baby units involve residential care setting. Very few have focused specifically on day units (18, 23–26). They describe the patient population and usually focus on the mother’s clinical improvement (27–31) or on the parental satisfaction (32, 33). The mean maternal age at the time of admission ranges from 24 (34) to 33 years (28) with a mean child age ranging from 9 weeks (29) to 7 months (35). The average length of stay ranged from 7 days [US study by (36)] to 11 weeks (37). The most frequent diagnosis for the mothers were depressive disorder, schizophrenia, or other psychotic disorders.

The second focus of these studies concerns the parent–child relationship which is assessed through video observations, attachment scales, or assessments of parental competences. In the literature, a significant improvement in the parent–child bonding (30, 38, 39, 40) or in the feelings of parental competence (31) is often described. Results are influenced by psychopathological and demographic factors (lower scores with a diagnosis of schizophrenia, personality disorder, lower social support or economic status) (29).

Few studies focus specifically on the well-being of the infants and on infant mental health (41). Other studies describe the symptomatology of the child at admission (29), the attachment type at discharge (42), or focus on the factors influencing the decision of child’s placement after admission (43, 44). This specific aspect needs to be considered, as the largest European prospective longitudinal study (43) shows that 14.8% of children were separated from their mother at discharge from mother–child baby units. Different risk factors were brought forward: medical complications for the baby, severe psychiatric disorders for mothers, severe psychiatric disorders for fathers, bad social relationships of the mother, disability for the mothers and low-social economic status.

There is a lack of international consensus on how these units should be structured and equipped (19, 45). Early identification of potentially at risk situations with a low outcome for the baby is therefore a major challenge.

Our study aims at contributing to this debate. In comparison with the literature dealing with day-night units, we will describe the care trajectory of the population of a parent-baby day unit, and focus more specifically on the baby’s status and on the parental psychopathology. The clinical evolution thanks to the day treatment, in terms of clinical improvement of the baby and the parent-baby relationship, will be evaluated at the time of discharge. The objective is to identify the specific variables related to a favorable or unfavorable outcome for the baby and to specify the clinical indications for day unit.

## Methods

### Description of the day unit

The Parent-Baby Day Hospitalization Unit (PBDH) opened its doors in 2015 within a tertiary pediatric hospital in Brussels. This innovative device is the first of its kind in the French-speaking part of Belgium. It is integrated in the child university psychiatry department and is in a great proximity with the pediatric department. It receives children from 0 to 2.5 years old, accompanied by their parents, on a part-time basis. The multidisciplinary team is composed of two child psychiatrists, a coordinator, two psychologists, a social worker, a psychomotrician, two pediatric nurses, three educators, two midwives and a secretary. A pediatrician and a psychiatrist are consultants at the request of the team.

The PBDH welcomes requests from parents or professionals regarding difficulties in the development of the child, functional disorders, difficulties in parenthood or in the establishment of early relationships. The capacity is 6 dyads/triads per day. Attendance can vary from 1 to 2 times per week. The Unit has a dual mandate of evaluation and therapeutic care. All admitted situations begin with an assessment period of 4 to 8 weeks.

The care is provided through group support and individual follow-up. During the day, different activities offer parental guidance, nursing assistance and a supportive environment for the child’s development and the relationship with the parent. Therapeutic individual interventions are based on multiple models (attachment, systemic, psychodynamic, and behavioral theories). Practitioners always rely on the developmental needs of infants as the basis for their guidance and on mentalization work with parents (46). The use of video is an integral part of the treatment as it can support parent’s reflection on their child and on their relationship with him/her. The unit tries to interact with the parents’ network, and to systematically meet the close family in contact with the child.

### Participants

All families admitted to the Parent-Baby Unit from its opening in 2015 to March 2020 were eligible for the study. Patients who attended the unit for only 1 day were excluded. All ethical measures regarding privacy, patient rights, and professional conduct were duly observed. This study was submitted to the HUDERF ethics committee, which gave its approval to start the study on 19/06/2020.

### Data collection

Data were collected from the child psychiatric medical records of the hospitalized patients.

Hospitalizations resulting in a discharge plan jointly supported by the parents, the team and the network are considered as “successful.” The hospitalization is considered “unsuccessful” when the child is placed or when developmental regression is observed (1 standard deviation below the mean for cognitive development between T1 and T2).

## Tools

### Child development and diagnosis

The Bayley Scale-III was used to **assess** child **development**. The *Bayley Scales of Infant and Toddler Development – BSID-III* assesses the development of young children between 1 and 42 months. The BSID-III is built around 5 scales: cognitive, language, motor, social–emotional, and behavioral. Only cognitive, language and motor scales were used here. The results for each scale are expressed as a composite score with a mean of 100 and a standard deviation of 15. We considered a score below 85 as a developmental delay.

**The relational withdrawal** was assessed with a clinical observation based on the ADBB (Alarm Distress Baby) scale developed by (47). The relational withdrawals were classified into 3 categories: no withdrawal, at risk of withdrawal and obvious withdrawal. Due to the low number of data collection, ADBB scores were not included in our study.

Regarding **the diagnostic assessment** of children, we have worked with the new edition of the Diagnostic Classification of Mental Health and Developmental Disorders in Early and Middle Childhood (DC: 0–5), (48). It is the updated and revised version of the DC: 0-3R classification.

**Physical health of the child** was evaluated by a pediatrician.

**Parent–child interactions** were evaluated using the Axis II (Relational Context) of the DC: 0–5 years old *scale (Zero to Three, 2005)* and more specifically the “Levels of adaptation of the parent–child relationship” scale. Four general levels of adjustment are described from level 1 (well-adjusted to satisfactory relationship) to level 4 (troubled to dangerous relationship). Parent–child interaction was assessed at T1 and T2 for each mother–child dyad.

**Mental and physical health of the parent** was considered either by the child psychiatrist in charge or by the consulting psychiatrist on the basis of the DSM V (49). Maternal depression was assessed using *The Edinburgh Postnatal Depression Scale (EPDS).* This scale is a 10-item self-administered questionnaire designed to assess the intensity of depressive symptoms experienced during the previous 7 days (50). A score of 12 or more is considered indicative of a risk of depression.

### Statistical analysis

The data drawn from the sample of the patients are used for descriptive and frequency statistics. Then, for the comparison of the variables of the two groups (successful hospitalization versus unsuccessful), the statistical tests are adapted to the type of variable. For continuous variables, the underlying assumptions of the *T*-test are tested (homogeneity of variances using the Bartlett test of homogeneity of variances and normality of residuals using the Shapiro-Wilks test). If the underlying assumptions are met, a *T*-test is made and the mean ± standard deviation is presented. Finally, we use a non-parametric test to compare the groups: the Wilcoxon rank test (*median* and inter-quartile range [*Q25 – Q75*] are presented). For discrete variables, we do the comparison using Pearson’s Chi^2^ test. A value of *p* <0.05 is considered significant and < 0.001 is considered highly significant.

## Results

### Admission and socio-demographic data

Ninety-two situations were admitted at the PBDH between May 2015 and March 2020. Data related to admissions and socio-demographic characteristics of our population are presented in Tables 1, 2.

**Table 1:** Admissions data.

	*N*	Mean (SD) or *N* (%)
Mission	92	
Evaluation group		18 (18.5)
Therapy group		74 (81.5)
Successful outcome		54 (73)
Unsuccessful outcome		20 (28)
Maternal age (years)		32.7 (6.3)
Paternal age (years)		36.3 (7.6)
Child age at admission (months)		14.8
0–6 months		31 (33.7)
6–12 months		12 (13)
12–24 months		26 (28.2)
>24 months		22 (23.9)
Child gender (percentage of girls)		40 (43.5)
Length of stay (week)		29
Evaluation group		7
Therapy group		34
Average frequentation	91	69.6
Reason for admission		
Concern for the child	92	83
None		9
Development delay		40 (43.5)
Withdrawal		16 (17.4)
Behavioral trouble		25 (27.2)
Somatic problem		2 (2.2)
Parent–child relational trouble	92	50 (54)
Parental difficulty	92	71 (77.2)
None		21 (22.8)
Parental skills		29 (31.5)
Acute psychiatric pathology		17 (18.5)
Chronic psychiatric pathology		25 (27.2)
Decision of the end of hospitalization (therapy group)		
Mutual decision		39
Parents		20
Medical staff		17
Partner support		
Involved in day care or appointments		18 (19.6)
Not present but supportive		27 (29.3)
Missing or lacking		47 (51.1)
Social or protectional service needed (yes)		30 (32.6)
Placement (yes)		16 (17.4)

**Table 2:** Socio-demographic characteristics.

	*N*	Mean (SD) or *N* (%)
Mother cultural origins	92	
Belgian		23 (25)
Maghreb		43 (46.7)
Sub-Saharan Africa		11 (11.9)
Eastern Europe		10 (10.9)
Europe (rest)		1 (1.1)
Turquey		3 (4.2)
South America		1 (1.1)
Father cultural origins		
Belgian		17 (18)
Maghreb		46 (50)
Sub-Saharan Africa		13 (14)
Eastern Europe		8 (9)
Europe (rest)		2 (2)
Turquey		4 (4)
South America		1 (1)
Lifestyle	92	
Couple		63 (68.5)
Isolated		20 (21.7)
Extended family		6 (6.5)
Parental center		3 (3.2)
Paternal recognition (yes)	91	74 (81.3)
Socio-economic level (family)	89	
Low		46 (51.7)
Middle		39 (43.8)
High		5 (5.6)
Familial support		
No		33 (36)
Low		12 (13)
Enough		47 (51)
Parity		
1		43 (46.7)
2		21 (22.8)
3		17 (18.5)
4 or more		11 (12)
History of child placement in the family	48	9 (18.8)
History of intervention of social service	48	15 (31.2)
History of medical or psychological antecedent in the siblings	48	30 (62.5)

In the majority of cases, babies and their family were referred for therapeutic management (81.5%) while the remaining (18.5%) were referred for assessment.

The mean parental age at admission was 32.7 years for mothers and 36.3 years for fathers. The mean age of children at admission was 14.8 months. The age distribution of children at admission is bimodal, with two peaks of attendance, around 7.2 and 25.3 months. A bit more than half of the children are admitted after 12 months (52.1%). The average length of hospitalization is 29 weeks, or 6.65 months (ranges from 1 week to 24.8 months) (1.6 months for the evaluation group and 7.8 months for the therapy group), with an average family attendance rate of 69.6%.

The reasons for admission were analyzed from the perspective of the baby, the parents, and the relationship. Each of these poles can constitute the reason, separately or jointly, for referral to the UPBB. Regarding the child’s reason for admission, there is a strong concern for the child himself (83%), the first reason being a developmental disorder (43.5%). Concerning the parent, in 77.2% of the cases, a maternal problem is the reason for the admission. Finally, for slightly more than half of the dyads/triads (54.3%), the parent recognizes a relationship disorder at admission.

The majority of referrals were from intern second line health care (40.6%) and from extern second line health care (28.6%). A smaller number of patients came directly from the front line health care (16.4%), from social services related to youth care or protection (8.8%) or directly on the patient’s initiative (5.5%). Nevertheless, 32.6% of the children had an open file with the youth care or youth protection (at the time of admission) and 8 children (8.7%) were in a placement situation (foster-care or intra-family).

The vast majority of admitted parents live together (68.8%). Most hosted families are of foreign cultural origin, with more than two languages spoken fluently at home (more than 75% in both cases), and half of them come from a disadvantaged socio-economic background. Only 3.4% of the mothers have an income as wage-earner, compared to 60% of the fathers.

Among families with more than one child, almost two-thirds have a history of medical or psychological follow-up for another child. One-fifth of families have had a court placement experience in their history, and one-third have had a social services intervention experience, whether it involves a parent or a child. 18.8% of the siblings had a history of placement and 31.2% had been followed by the youth protection services. The vast majority of families with more than a child (62.5%) had a psychological or somatic history for the children.

Concerning the involvement of fathers, he is absent half of the time (51.1%). He is not involved but supportive in 29.3% of cases and present in 19.6% of cases.

### Pregnancy, neonatal, and child data

Pregnancy and Infant characteristics are presented in Table 3.

**Table 3:** Pregnancy and infant characteristics.

	*N*	Mean (SD) or *N* (%)
Pregnancy desire (yes)	92	79 (85)
Medically assisted procreation	92	6 (6.5)
Miscarriage antecedent	88	29 (32.9)
Stress during pregnancy	92	60 (65.2)
Tobacco during pregnancy	88	15 (17)
Drugs during pregnancy	87	9 (10.3)
Psychiatric treatment	92	6 (6.5)
Delivery mode	90	
Cesarean		21 (23.3)
Vaginal		69 (76.7)
Traumatism at delivery	81	25 (30.9)
Stay in neonatalogy	91	31 (34)
Breastfeeding	91	59 (64.8)
Weight < 2.5 kg at birth		24 (26.1)
Growth		
Normal		72 (78.3)
Upper the curve		8 (8.7)
Under the curve		12 (13)
Gestational age / prematurity		
Term		61 (66.3)
Late prematurity		20 (21.7)
Moderate		5 (5.4)
High		3 (3.3)
Extreme		2 (2.2)
Infant mental health diagnosis (DC 0–5)		
No diagnosis		23 (25)
Neuro-developmental disorder		24 (26)
Sensory processing disorder		2 (2)
Anxiety disorder		18 (19.6)
Mood disorder		9 (9.8)
Sleep disorder		18 (19.6)
Eating disorder		9 (9.8)
Related to trauma		19 (20.6)
Specific relation disorder		9 (9.8)
Somatic problems		42 (45.6)
Witness to domestic violence		35 (38)
Neglect		32 (34.8)
Maltreatment		13 (14.1)

More than 25% of the children were born premature [statistical difference with the national average of 8% (51)] and just over a quarter of the children were born with low birth weight (26.1%). In comparison, the national low birth weight rate is 7.8% (value of *p* = <0.001) (CEPIP).

Concerning the antenatal period, two thirds reported stress factors during pregnancy and one third of the mothers had a history of miscarriage, a traumatic delivery and/or a hospitalization in the neonatal intensive care. One-fourth (23.3%) of the deliveries was made by cesarean section, this proportion is not significantly different from the Belgian national rate.

In terms of drug use, nearly one-fifth of the mothers smoked during their pregnancy, one-tenth used drugs and 6.5% were under psychiatric treatment.

Nearly half of the children admitted had a somatic or functional pathology at the time of admission, mainly gastroenterology pathology, sleep disorder (20%), and eating disorder (15%).

The diagnosis of the children at admission, according to the DC 0–5 scale (Axis I) shows that 26% of the children present a developmental disorder (global developmental delay, language delay, ASD), 19.6% present an anxiety disorder (generalized, separation anxiety) and 20.6% present symptoms related to a trauma.

The developmental scales show that more than a quarter of the children (26.1%) are admitted with a cognitive delay (scale score below 85), slightly less than a half (47.7%) with a language delay and 20% with a motor delay. Children also presented high risk (28.1%) or clinical withdrawal (39.3%) at entrance.

The children witnessed domestic violence in 38% of the situations and have also experienced separation from their attachment figure or have been neglected. In 34.8% of cases, children experienced neglect and 14.1% abuse.

### Child development and clinical improvement

**Figure 1:**
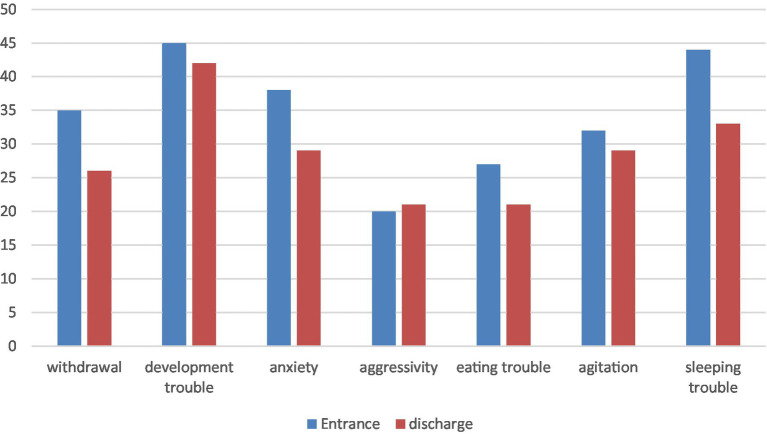
Clinical evolution between entrance and discharge.

Results at the Bayley and symptoms at entrance and discharge are presented in Table 4 and Figure 1. Sixty-five children received the developmental assessment at entrance and 26 where evaluated at the end of the intervention. An improvement in the developmental quotient between entry and discharge is observed but it does not reach statistical significance.

**Table 4:** Comparison of child development, withdrawal and parent–child interaction between entrance and discharge.

	Entrance	Discharge
Bayley (QD)	(*n* = 65)	(*n* = 26)
Cognitive	90.3 (26.1% <85)	97.3
Communication	85.9 (47.7% <85)	89.6
Motor	91.9 (20% <85)	97.5
Clinical withdrawal	(*n* = 89)	(*n* = 89)
No	29 (32.6%)	44 (49.4%)
At risk	25 (28.1%)	19 (21.3%)
withdrawal	35 (39.3%)	26 (29.3%)
Relational level	(*n* = 91)	(*n* = 91)
1	12 (13.2%)	28 (30.8%)
2	38 (41.8%)	26 (28.6%)
3	37 (40.6%)	32 (35.2%)
4	4 (4.4%)	5 (5.4%)

With respect to psychopathological symptoms, each child presents an average of 2.9 symptoms at admission, with an overrepresentation of symptoms of developmental disorder, sleep disorder, separation anxiety and relational withdrawal. At discharge, the children presented a symptom reduction with an average of 2.4 symptoms and a reduction of child’s withdrawal but it does not reach statistical significance.

### Parent–child interaction improvement

Nearly all patients have significant relational parent-baby relationship problems at admission. At entry, according to axis 2 of the DC 0–5, 41.8% of the dyads/tryads have type 2 relationships (strained to concerning) and 40.6% have type 3 relationships (compromised to disturbed). 13.2% have a relationship described as adapted appropriately and satisfactory (type 1). At discharge, 28.6% of patients had a type 2 relationship, 35.2% had a type 3 relationship and 30.8% had a type 1 relationship. These results are presented in Table 4.

### Parental data

Mother and father clinical characteristics are presented in Table 5.

**Table 5:** Parental clinical characteristics.

	*N*	Mean (SD) or *N* (%)
Mothers characteristics		
Suicide attempt antecedent	91	17 (18.6)
Psychiatric follow-up at entrance	91	49 (53.8)
Somatic problem	90	38 (41.3)
History of physical or psychical trauma	86	44 (51.2)
Edinburgh scale	23	17
DSM V diagnostic	92	
No diagnostic		13 (14.1)
Schizophrenia/non affective psychosis		4 (4.3)
Depressive disorder		52 (56.5)
Anxiety disorder		24 (26.1)
Borderline personality disorder		24 (26.1)
Trauma disorder		6 (6.5)
Eating disorder		3 (3.2)
Substance use disorder		10 (10.9)
Bipolar disorder		1 (1.1)
Neurocognitive disorder		7 (7.6)
ASD		0
Conduct disorders		0
Comorbidity		61
Fathers characteristics		
Suicide attempt antecedent	65	2 (3.1)
Psychiatric follow-up at entrance	66	5 (7.5)
Somatic problem	66	15 (22.7)
History of physical or psychical trauma	61	10 (16.4)
Edinburgh scale		/
DSM V diagnostic	62	
No diagnostic		37 (59.7)
Schizophrenia/non affective psychosis		2 (3.2)
Depressive disorder		8 (12.9)
Anxiety disorder		3 (4.8)
Borderline personality disorder		7 (11.3)
Trauma disorder		5 (8.1)
Eating disorder		0
Substance use disorder		8 (12.9)
Bipolar disorder		0
Neurocognitive disorder		3 (4.8)
ASD		1 (1.6)
Conduct disorders		4 (6.4)

Among the mothers admitted to the PBDH, 53.8% have a psychologist or psychiatric follow-up.

The average score obtained for the Edinburgh Scale was 17, indicating the presence of depressive symptoms and a high risk of depression for these mothers. This result can be linked to the mothers’ diagnoses according to axis 1 of the DSM V: 52 have a depressive pathology (56.5%), 24 show anxiety (26%) and 24 have a borderline pathology (26%). The majority of the mothers in our care unit are suffering from anxiety and depression, with significant comorbidity (66.3% of the mothers have at least two diagnoses).

It should be noted that 41.3% have somatic problems and that more than one mother out of two (51.2%) have a traumatic past (history of psychological or physical abuse).

Concerning the fathers, 22.7% of them present a somatic pathology. 16.4% of the fathers have a traumatic past, as well as the same pathologies than the mothers (according to axis 1 of the DSM V) but to a lesser extent: depressive and borderline pathologies. This can be explained by a lack of comprehensive data collected from the fathers.

### Factors contributing to a positive change in hospitalization

Table 6 examines the variables that could influence the outcome of the hospitalization. Regarding the impact of the family context, the mother’s age at admission has a significant influence on the outcome of the hospitalization. It is not the case for fathers. When the hospitalization is successful, the mothers are on average 33.1 years old at admission (±6.16) against 29.4 years old (±6.21) when the hospitalization is not successful.

**Table 6:** Analysis of the variables influencing the outcome of the hospitalization.

Variable	Unsuccessful (*n* = 20)	Successful (*n* = 54)	value of *p*
Mother’s age	29.4 ± 6.21	33.1 ± 6.16	<0.05
Father’s age	33 [30.75–34.25]	33.5 [29–41]	0.44
Socio economic level			0.07
Low	15 (75%)	23 (42.59%)	
Middle	5 (25%)	25 (46.3%)	
High	0 (0%)	4 (7.41%)	
Unknown	0 (0%)	2 (3.7%)	
Familial support			0.30
No	10 (50%)	18 (33.33%)	
Low	1 (5%)	8 (14.81%)	
Enough	9 (45%)	28 (51.9%)	
Reason of admission			
Relational trouble	12 (60%)	35 (64.9%)	0.91
Parental difficulty			<0.05
Parental skills	6 (30%)	18 (33.33%)	
Chronic psych. Path.	10 (50%)	15 (27.8%)	
Acute psych. Path.	0 (0%)	17 (31.48%)	
None	4 (20%)	4 (7.41%)	
Concern for the child			0.07
Behavior	4 (20%)	17 (31.48%)	
Development delay	12 (60%)	15 (27.78%)	
Withdrawal	4 (20%)	12 (22.22%)	
Somatic problem	0 (0%)	2 (3.7%)	
None	0 (0%)	8 (14.81%)	
Average frequentation (d/w)	0.72 [0.55–0.86]	0.68 [0.56–0.85]	0.94
Length of stay (weeks)	27.5 [13.75–50.5]	26.5 [12.5–49]	0.83
Partner support			0.04
Involved	6 (30%)	7 (12.97%)	
Supportive	1 (5%)	16 (29.63%)	
Missing or lacking	13 (65%)	31 (57.41%)	
Youth care or protection entrance			<0.01
0	6 (30%)	40 (74.07%)	
Youth care	7 (35%)	9 (16.67%)	
Youth protection	7 (35%)	5 (9.26%)	
Low birth weight			0.9111
0	15 (75%)	43 (79.63%)	
1	5 (25%)	11/ (20.37%)	
Witness to domestic violence			<0.01
0	4 (20%)	36 (66.67%)	
1	16 (80%)	18 (33.33%)	
Neglect			<0.001
0	1 (5%)	44 (81.48%)	
1	19 (95%)	10 (18.52%)	
Maltreatment			<0.001
0	10 (50%)	52 (96.3%)	
1	10 (50%)	2 (3.7%)	
ADBB score at entrance	10.75 ± 5.5	7.26 ± 5.11	0.31
Bayley score at entrance			
Cognitive score	89.06 ± 15.4	91.48 ± 16.8	0.61
Communication score	80.38 ± 12.3	90.51 ± 16.78	<0.05
Motricity score	85.25 ± 14.23	94.89 ± 15.68	<0.05
Child diagnostic at entrance			<0.05
0	2 (10%)	19 (35.19%)	
Sensory processing disorder	0 (0%)	2 (3.7%)	
Mood disorder	2 (10%)	5 (9.25%)	
Related to trauma	10 (50%)	4 (7.41%)	
Specific relational trouble	1 (5%)	1 (1.85%)	
Autism spectrum trouble	0 (0%)	2 (3.7%)	
Global developmental delay	3 (15%)	1 (1.85%)	
Language delay	1 (5%)	1 (1.85%)	
Other neuro-developmental	0 (0%)	1 (1.85%)	
Separation anxiety	0 (0%)	10 (18.52%)	
Generalized anxiety	1 (5%)	3 (5.56%)	
Sleeping trouble	0 (0%)	4 (7.41%)	
Eating trouble	0 (0%)	2 (3.7%)	
Relational level entrance (Axis II DC 0–5)			<0.01
1	0 (0%)	1 (1.89%)	
2	3 (15%)	32 (60.38%)	
3	15 (75%)	19 (35.85%)	
4	2 (10%)	1 (1.89%)	
Sibling placement history (*n* = 10)	6/10 (60%)	2/26 (7.7%)	<0.01
Maternal suicide attempt	6 (30%)	10 (18.5%)	0.4547
Father’s suicide attempt	0	1 (1.8%)	1
Maternal trauma history	19/19 (100%)	23/49 (46.9%)	<0.001
Father trauma history	4/11 (36.3%)	5/27 (18.5%)	0.30
Maternal diagnostic (DSM V)			0.048
0	0/20 (0%)	3/53	
1	1/20 (5%)	3/53	
2	9/20 (45%)	27/53 (50.94%)	
3	0/20 (0%)	11/53 (20.75%)	
4	3/20 (15%)	5/53	
5	2/20 (10%)	2/53	
7	4/20 (20%)	2/53	
9	1/20 (5%)	0/53	
Paternal diagnostic			0.1077

The age of the children at admission has no significant influence on the outcome of the hospitalization, following the Wilcoxon test.

Neither family support (*p* = 0.30) nor socioeconomic level (*p* = 0.07) seems to have a significant influence on the outcome of hospitalization.

Concerning the setting of the hospitalization, the Mc Nemar’s Chi^2^ test highlights that the reason for hospitalization on the mother’s side significantly influences the outcome of the hospitalization (*χ*^2^ = 10.62, value of *p* = 0.01). On the contrary, the reason for admission on the child’s side, the rate of attendance and the duration of hospitalization do not significantly influence the outcome of hospitalization.

Clinical situations in which mothers got support from their partners regarding the hospitalization (*χ*^2^ = 6.40, value of *p* = 0.04) without their partners being present in the care at the PBDH, have a 94% rate of success. This ratio drops to 54% when the partner is present and to 70.5% when they are absent and do not support the process. Absent partners represent 65% of the “unsuccessful” group.

Finally, the presence of a legal framework such as the youth care or youth protection at admission (*χ*^2^ = 12.79, value of *p* = 0.0001) significantly influences the outcome of the hospitalization. Hospitalizations made outside of a legal framework at admission have a positive outcome in 86.95% of the cases, whereas this ratio drops to 56% when children are admitted through a youth care and 42% through a youth protection service.

For variables grouping child-related data, those who witnessed domestic violence (*χ*^2^ = 10.98, value of *p* < 0.001), experienced neglect (*χ*^2^ = 32.68, value of *p* < 0.001) or abuse (*χ*^2^ = 19.74, value of *p* < 0.001) are more represented in the “unsuccessful hospitalization” group. There is therefore a significant influence on the outcome of hospitalization for these three variables.

Regarding child development, the mean scores at admission for the language scale and the motor scale have a significant influence on the evolution of the hospitalization. When hospitalization was successful, children had a mean score of 90.51 (±16.78) on the language scale, compared with 80.38 (±12.3) for those whose hospitalization was not successful. The children obtained an average score of 94.89 (±15.68) on the motor scale, compared with 85.25 (±14.23) for the children belonging to the “uncompleted hospitalization” group.

The child’s diagnosis (according to DC: 0–5) also has a significant impact in the outcome of hospitalization (*χ*^2^ = 33.68, value of *p* = 0.001). Trauma-related disorders are more represented (50%) in the “unsuccessful hospitalization” group and account for 1 in 2 patients, whereas separation anxiety disorder is the most represented (18.52%) in the “successful hospitalization” group. Nevertheless, the absence of disorder is the most represented (35.19%) in the successful hospitalization group.

Relationship quality is closely linked to the outcome of hospitalization at PBDH (*χ*^2^ = 13.71, value of *p* = 0.003). The vast majority of patients present a type 3 relationship (compromised to disturbed) in the “unsuccessful hospitalization” group, whereas the type 2 relationship (strained to concerning) represent 60% of the “successful hospitalization” group. Patients with the latter type of relationship have 91.4% (32/32 + 3) chances to have a successful hospitalization. This ratio drops to 55.9% (19/19 + 15) for type 3 relationships. Finally, the Wilcoxon test suggests that there is a significant improvement in the parent-baby relationship between entry and discharge when the hospitalization is successful (*p* < 0.001).

Regarding siblings, a history of placement significantly influences the outcome of hospitalization. When the hospitalized child’s siblings have been placed, the hospitalization has a 25% probability to succeed. The number of siblings with a history of placement is more represented in the “unsuccessful hospitalization” group (60%).

With respect to parental history variables, there was significantly (χ^2^ = 14.15, value of *p*<0.001) more traumatic history among mothers in the “hospitalization not completed” group (100%). This feature is not found among the fathers. The maternal pathology significantly influences the outcome of the hospitalization. Depressive disorders (2; 50.94%) and anxiety disorders (3; 20.75%) are more represented in the “completed hospitalization” group. Depressive disorders are also found in 45% of cases in the “not completed” group and are the most common disorder in this latter group.

## Discussion

This retrospective study is the first major study to focus specifically on the developmental and clinical improvement of the baby in a PBDH. It is also the first survey to highlight significant differences between day and day/night units, both in the population data and in the characteristics of admission in a parent-baby unit. Strengths and limitations will be discussed.

### Admission data

One of the first findings is that the length of stay in day units (29 weeks) is much longer than in day/night facilities. Based on the existing literature, the durations of the stay in the later vary from 5.93 days for a study in the US (36) to 11.6 weeks in Israel (37). It is even more obvious when considering only therapeutic care (34 weeks). These differences for day/night units seem to partly result from the different nature and purpose of MBUs in different countries and regions. Nevertheless, the possibility of receiving intensive care in a day unit, avoiding the break in contact inherent in a day/night hospitalization, undoubtedly offers a longer-term perspective of therapeutic management. Care is also longer for chronic pathologies and personality disorders (22), yet our sample includes a high rate of mothers with borderline personality disorder (26%). Based on the type of psychopathology we receive (including a high rate of parents who have experienced traumas weakening the construction of the bond with the other), the high duration of the care seems necessary to accompany these families properly and respect their temporality. It is also necessary to consider that the hospitalization framework, putting the baby at the very heart of the care, has an influence on the duration of hospitalization. Clearly, the outcome changes if the objective of hospitalization is the clinical improvement of the parent or of the baby.

The average age of care in day units differs from that of full-time units, where most admissions take place very early (before 8 weeks of age). The greater psychiatric morbidity of mothers admitted to residential units probably explains the earlier age of admission (52). The high percentage of mothers suffering from depression is a factor that probably contributes to a later age of admission (average age 15 months). It might indeed literally immobilize the mother (interest in home care), and present constraints related to traveling with a young child. These results should encourage the development of psychiatric services offering postpartum home care. Furthermore, the positioning of university hospitals as third-line hospitals may also explain the later move of families who already benefit from outpatient care before being reoriented to day hospitalization. Finally, in many day/night units, the age of admission for babies is sometimes limited to the first year of life, whereas our system welcomes them until they start kindergarten.

Unlike the situation in French MBUs, where the number of voluntary admissions reaches 90%, in our PBDH a significant number of admissions are supported or imposed by the youth care or the youth protection. In our study, 32.6% of the families are supervised by one of these two services. This percentage is similar to the one established during the Marcé research in Belgium between 2001 and 2007 (34%) (53). The ways in which medical and social services work together differ significantly in the two countries.

Examination of the grounds for admission shows a significant difference from what is described in the literature for day/night units. The results of the Marcé study show that in 80% of cases, “children are generally well” on admission. In our study, this proportion is reversed, since in 90.2% of cases, difficulties for the baby are observed on admission (all areas of development combined). Once again, it seems that one explanation for this situation is the one of the positioning of the facility, which the workers identify as a “care structure at the baby’s departure,” whereas the day/night mother-baby units rather welcome mother populations identified as being at risk, in an earlier way at the end of the maternity ward when the baby’s psychopathology is not yet at the forefront.

The high rate (17.4%) of referral of the baby to a care facility at the end of the hospitalization deserves attention, since it is similar to or exceeds certain percentages found in day/night studies in Europe [4 out of 23 placements, i.e., 17.4% in the study by (54); 14.8% in the study by (43)]. It should be taken into consideration that some of the children (*n* = 7) were already in institutional care (nursery or hospital) and that 5 of them remained there after hospitalization. The higher average age of our population probably partly explains this high rate since a decision for separation is sometimes taken when successive therapeutic proposals fail. It is interesting to note that in other recent studies, including an Australian study examining the orientation of patients at discharge from hospital (55), the return of children to their home with their parent (s) is almost systematic (only 2.4% separation), raising questions involving the health and child protection policies in place in each country.

### Social vulnerabilities, partner involving, and familial support

As we expected, the context of vulnerability is similar in our population and in the day/night units. The vast majority of parents have poor financial resources, lack family or extra-familial assistance and have a family history of immigration. These data are difficult to compare with the literature given the heterogeneity of the measures used.

Nevertheless, it is imperative to consider the seriousness of this vulnerability, since it will be added, for some parents, to psychopathology and/or traumas experienced in childhood. The interplay between socioeconomic factors, migration history and perinatal health is widely described in the literature (56, 57). However, unlike what has been shown in previous studies (58), none of the economic or social variables considered individually, is associated with a pejorative outcome of hospitalization in our study. Only the mother’s age seems to have an influence on the outcome of the hospitalization. Indeed, early motherhood has been widely shown to be associated with poorer child development and young mothers are at increased risk for postpartum depression (59, 60). Multivariate statistical analyses could help measuring the risk associated with cumulative factors. We have also studied data related to the lifestyle of the families in care. As shown in other studies on MBUs, the majority of families attending the PBDH live together as couples [68.5% in our study vs. 68–90% according to the studies reviewed, (24)], yet we expected to receive a majority of single parents since support by the group is an indication for referral to our service.

The place of fathers in mother-baby hospitalization and perinatal care facilities has evolved considerably since the units first opened (61), but this topic is hardly addressed in the research literature (62, 63). Nowadays, some residential units have small studios or rooms set up to accommodate inpatient couples. The name “Parent-Baby Day Unit” implies, *de facto*, the inclusion of the partner in the care. In our study, the isolation of a large number of women in the exercise of their parental functions was found (absence of the father or of his support in 51% of the cases). The comparison of the two populations shows that hospitalization is most likely to be successful when the father is supportive, without necessarily being present at the hospital. A more detailed analysis of these data helps understanding this paradox since paternal psychopathology concerned a higher number of fathers when the father was the caregiver involved with the baby during hospitalization (15 of 18). These results should be considered with caution, since a considerable amount of data concerning paternal psychopathology is missing. In addition, it was easier to assess parental psychopathology for fathers who spent full days in the unit. In any case, these data support the point of view of some authors who consider the father’s refusal of any contact with the hospitalization services as a poor prognosis factor for the evolution of the dyad (44).

### Focus on the child and the pregnancy

The analysis of the perinatal risk factors in our population illustrates the importance of antenatal prevention: most of the families attending our unit have a history of complicated pregnancy or immediate postpartum. The high proportion of children who stayed in the neonatal unit (34%) and the number of children with low birth weight (26.1%) clearly highlights it. This latter rate is statistically significantly higher than the data in the Belgian population, and the literature has highlighted its correlation with socioeconomic risk factors like in our population (64). The study by Wright et al. (41) similarly found an abnormally high rate of children with low birth weight (20%) among children admitted in a MBU, while the percentage of prematurity did not differ from their national average. As a reminder, many epidemiological studies show a link between major psycho-emotional stress during pregnancy or low birth weight and subsequent outcomes in terms of psychopathology but also cardiovascular diseases (65, 66).

The major results of this research show an improvement in the developmental quotient of the child between entry and discharge. There is also an overall decrease in the symptomatology present at entry. None of these results is however statistically significant and it must therefore be interpreted with caution given the inherent bias of the retrospective aspect of this study. A major weakness of this work was the difficulty to collect quantitative data. Developmental scales could only be collected at discharge for 26 children (compared to 65 at entry). There are three possible reasons for this loss of data. The main one is that the duration of intervention for many children (9) did not allow for re-administration of the test (the test is administered at 6-month intervals to avoid learning bias). The second reason is linked to an abrupt interruption in the care process with no test at the end (generally decided by the parents) in 9 situations. Finally, the last explanation is the difficulty of collecting quantitative data from a research perspective while engaged in a clinical process (e.g., systematizing the use of exit scales even if the patient is clinically better) (9 situations). Equally, the improvement in scores cognition, language could have been better studied if the developmental assessment had been done in relatively older age groups (the mean age of admission of the infant was 14.8 months, one third of the population was below 6 months and around 50% was less than one year). This is given to lower scores of reliability of Bayley scale in younger age groups (67). Similarly, for showing a visible change in scores in Bayley scale with any intervention a longer period of interval (T2−T1) is preferable. In this study, the average period was 6.65 months (range from 1 week to 24.8 months).

There is a lack of use of a validated grid to collect symptoms. The age range of the infants at admission was lower in the use of Pediatric behavioral assessment scales or check lists though some infants in the present study were referred with behavioral trouble. Using an appropriate scale for sensory integration profile could be an important parameter for assessment in infants who have suffered from stress disorders, since around 30% infants were found to have suffered from sleep and eating disorders in the present study. But, at this time, the most significant test (Infant/Toddler sensory profile instrument) is still not translated and validated in French for this age group (68).

Nevertheless, this is the first study to systematically examine the development of children in a day parent-baby unit using a standardized developmental scale. Only two other studies conducted on parent-baby day-units look at the psychopathology and development of the child at entry (29, 41). The first used the Marcé questionnaire and reported the following figures (child’s condition at entry): 76% cases children in good health, 11% psychomotor problems, 9% emotional problems and 5% somatic problems. These results are far from ours, which reveal an alarming picture of the children’s condition at entry in our unit (45.6% somatic problems, 26.1% cognitive delay, 47% communication delay and 20% motor delay). Concern for the child’s condition is even greater in the unfavorable outcome group (significative statistical difference for communication and motricity assessment). The second study (41) used the Ages & Stages Questionnaires-3 to screen children’s development, and found the same trends than us, with greater difficulties in the socio-emotional areas than in the motor and cognitive areas. The strong representation of somatic problems, and in particular gastro-alimentary problems, should encourage the team to propose more targeted therapeutic treatments for this group of pathologies (sensory workshops around food, more individual care during meals, cooking workshops with parents, integration of a speech therapist in the therapeutic team, etc.)

Another study looks at the early relational withdrawal of children from mothers hospitalized in MBU. The study boosts it around 35% (69). We find in the PBDH the same proportion, 39.3% of children with marked relational withdrawal, to which can be added those who are considered at risk of withdrawal (28.1%). Despite the fact that number of children admitted to the unit for developmental assessment show withdrawal and communication disorders, this proportion remains significant. In the general population, a high prevalence (up to 75%) is found among infants whose mothers met criteria for a major depressive episode at 6 months postpartum (70).

The child population at PBDH is often impacted by trauma and the results show that these life events significantly influence the hospitalization negatively. The rates of neglect and the number of children witnessing domestic violence are very high (34.8 and 38% of cases respectively), just as the number of children presenting disorders related to mistreatment and overrepresented in the pejorative group. In comparison, data from the literature estimate neglect at 1–15%, and witnessing domestic violence at 10–20% (71).

### Dyadic relation and parental psychopathology

The assessment of the relationship through the Axis II of the 0–5 years scale shows an improvement of the relationship between the partners of the mother–child dyad at the end of the care process. This improvement is also statistically higher in the successful group and the dyadic relation was worser in pejorative group, as expected. Although the 0–3 scale is recognized for the value of its multiaxial assessment, (72) point out the lack of a quantitative criteria between pathological and non-pathological, as well as the lack of information related to the degree of intensity of the symptom and explanation of its meaning. Consequently, the parent–child interaction should be investigated through other tools at PBDH. It would be interesting to know the relational attachment patterns of parents and children.

Compared to the populations in MBUs, the psychopathology of the parents appears to be less severe in the PBDH unit. The maternal population is mainly represented by depressive disorders (37%) and anxiety disorders (26%), whereas a systematic review of MBUs identifies depressive disorders (50%), psychotic disorders (25%), and bipolar disorders (10%) as the most frequent pathologies. (24). Anxiety disorders are therefore much more represented in PBDH than in UMBs, which had been highlighted by the study of a day unit (73). High anxiety levels are known to be possible risk factor for the development of disorders in parenting (74). The high rate of trauma experienced by the patients was predictable, given the psychiatric fragility of the sample population, and the prevalence of borderline personality disorder (29%), as shown in previous studies of MBU’s (75). The appropriate management of this pathology is even more important given that a study identified it as one of the first factors associated with placement in a population attending a mother-baby unit (76). Our results did not show a significant influence of this pathology on the outcome, but maternal trauma history is over-represented in our pejorative evolution group. For the management of this disorder, scientists generally advocate a therapeutic setting that combines flexibility, stability, and availability (77), which characterizes daytime settings over a day/night structure.

Interestingly, our study shows statistical differences in the repartition of psychopathological pathology between the two groups. Mothers with an anxious diagnosis are all included in the successful group. This suggests that this profile of patients benefits particularly from our care.

Parents’ somatic complaints are seldom studied in the literature. Our study shows that this aspect of parental symptomatology is significant (39% of mothers, 26% of fathers show a current somatic pathology) and must be addressed simultaneously within the care to the families (collaboration with a treating physician, relay to the proximity care network, workshops or body therapies) because it can sometimes be the only witness of a parent’s psychic suffering (psycho-somatic disorder). The need to work hand in hand with a psychiatrist and a pediatrician is obvious in the perinatal care system, but the aspect of the parents’ physical health is insufficiently addressed.

### Conclusion and perspectives

This study contributes to giving the baby its central place in perinatal settings, particularly in mother-baby units where concern for the mother was historically the primary focus of care. The strength of our research lies in the precise description of the physical and developmental state of the baby at admission to a parent-baby day unit. Developmental, behavioral, and relational improvement was observed at discharge but the sustainable effect of intervention needs prospective long term follow up studies.

The differences observed in our study between the day/night population and the daytime population should contribute to improve the care pathway for families. In our study, the system seemed to be more effective for anxiety disorders of the child and the parent. It also underlines the vigilance necessary for the implementation of care which could be inadequate for the child when his clinical presentation is already worrying at the time of admission.

## Data availability statement

The raw data supporting the conclusions of this article will be made available by the authors, without undue reservation.

## Ethics statement

The studies involving human participants were reviewed and approved by Comité d’éthique de l’hôpital universitaire des enfants Reine Fabiola. Written informed consent to participate in this study was provided by the participants’ legal guardian/next of kin.

## Author contributions

VD conceptualized the study, and wrote the grant funding proposition, together with AM, LC, and CG. AM prepared the first draft and subsequent versions of the protocol and this manuscript, wrote the ethical approvals documents, and selected the parental scales. AM and LC collected research data and analyzed them. LC and CG are the psychologists responsible for the developmental and relational assessment tools. PB was statistician who led the design of statistical analysis model. All authors contributed to the article and approved the submitted version.

## Conflict of interest

The authors declare that the research was conducted in the absence of any commercial or financial relationships that could be construed as a potential conflict of interest.

## Publisher’s note

All claims expressed in this article are solely those of the authors and do not necessarily represent those of their affiliated organizations, or those of the publisher, the editors and the reviewers. Any product that may be evaluated in this article, or claim that may be made by its manufacturer, is not guaranteed or endorsed by the publisher.
